# Street-level imagery dataset for the detection of informal vendors in urban environment

**DOI:** 10.1016/j.dib.2025.111912

**Published:** 2025-07-20

**Authors:** Keyla Garcia-Jaimes, John R. Ballesteros, John W. Branch-Bedoya

**Affiliations:** Departamento de Ciencias de la Computación y de la Decisión, Facultad de Minas, Universidad Nacional de Colombia, Medellín 050041, Colombia

**Keywords:** Informal economy, Action camera, Parallel economy, Deep Learning, GeoIA, Public policy, Neuronal networks

## Abstract

Street vending is a prominent component of the informal economy, yet its prevalence remains poorly quantified due to the limitations of traditional survey methods, which are costly, invasive, and labor-intensive. To enable scalable, image-based assessments of this activity, we present the StreetVendor-SLI dataset, specifically designed for detecting vendors in urban environments. The dataset comprises 2794 high-resolution images (2416×1359 px), obtained from video footage recorded with a user grade camera mounted on a motorcycle. The original dataset contains 1397 images, with an average size of 5 MB per image, resulting in a total dataset size of 4.63 GB. Privacy compliance with GDPR guidelines was achieved by anonymizing pedestrian faces and vehicle license plates using an open-source YOLO object detection pipeline. Every image is annotated utilizing the YOLO format, with vendors enclosed in bounding boxes and classified into three categories: fixed-stall vendor (1774 labels), semi-fixed vendor (459 labels), and itinerant vendor (124 labels). To address class imbalance and enhance model generalization, data augmentation techniques—including geometric transformations (rotation, flipping, scaling, shearing) and spectral adjustments (brightness, contrast, hue)—were applied. The Steet-level Imagery dataset thus provides an openly available option for the detection of street vendors, offering a valuable resource for researchers studying informal economic activities and urban policies.

## Graphical abstract

**Figure:**
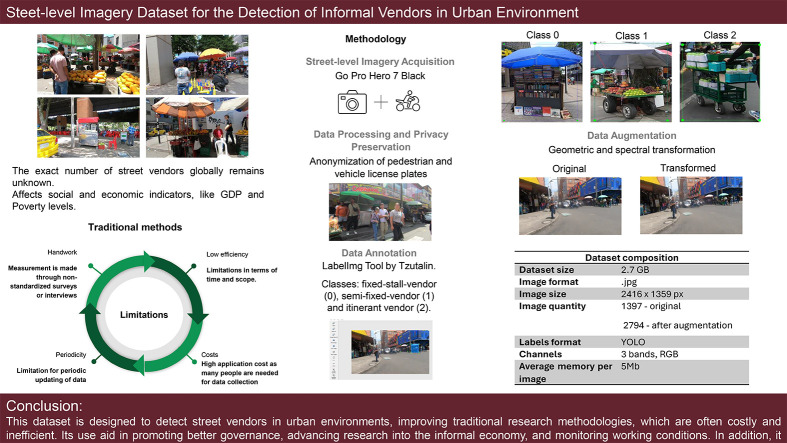

Specifications Table

**Table:** 

Subject	Computer Science, Artificial Intelligence and Applied Machine Learning
Specific subject area	Street-level Imagery (SLI) collection for detecting informal vendors in urban environments using deep learning
Type of data	Images in *.jpg format Labelled Dataset in YOLO format
Data collection	Side-looking videos were captured using a GoPro Hero 7 Black camera mounted on a motorcycle handlebar. A Python script processed the video into street-view images, extracting one SLI frame every two seconds. Data collection involved traveling 37 km across 10 transects in downtown of Medellin-Colombia, a Latin American city known for its high concentration of street vendors. Each trip lasted 30–35 min, with two batteries swapped as needed. Recordings were conducted in daylight between 9:00 AM and 12:00 PM.
Data source location	City/Town/Region: Medellín/La Candelaria/Antioquia. Country: Colombia
Data accessibility	Repository name: Zenodo. [1] DOI link: https://doi.org/10.5281/zenodo.14635548
Related research article	None

## 1 Value of the Data

• Understanding the scale of street vending is critical for accurately assessing its economic contribution, evaluating labour conditions and quality, promoting legal and social protections for workers in this sector, and developing effective regulatory and policy frameworks [2].
• Measuring street vending employment is challenging. Street vendors form a large, mobile, and dynamic group whose businesses are dispersed across urban areas. The above makes difficult the data collection through conventional methods and sources like surveys and interviews [3,4].
• Identifying the street vendors is key to improving governance and advancing geographical research on informal economies. Mapping this sector supports offering critical insights into how these activities intersect with urban socio-economic and physical environments. Resultant insights are essential for shaping regulatory policies, driving reforms, and guiding urban development strategies [3].
• Despite the need for innovative approaches to data collection in this sector it remains as a notable shortage of datasets for training neural networks to detect street vending employment.
• This dataset provides an example of a pipeline for creating additional datasets using grade action cameras, offering an alternative to proprietary platforms like Google Street View. Unlike open or crowd-sourced street-level imagery, which often lacks quality and privacy, this dataset ensures high-resolution, anonymized content tailored for detecting and analysing informal sales activities. Moreover, recent changes to Google's API pricing and terms of use highlight an urgent need for open-source, researcher-friendly solutions, making this dataset a valuable resource for advancing studies in this domain [5,6].
• The dataset offers insights for scholars and urban researchers studying the economic impact of street vending, vendor mobility patterns, and their interactions with formal markets. It also facilitates the analysis of social dynamics, such as the link between informal economies and urban socio-economic inequality, while supporting urban studies on the spatial distribution and density of street vendors. This can guide urban planning, zoning, policies and strategies aimed at integrating or regulating informal economies within the cities.

## 2 Background

Informal employment, particularly street vending is an important part of the economy in the development countries. Whoever, it remains difficult to quantify due to vendors' migration, spatial dispersion, and exclusion from conventional survey methods. Household questionnaires and in-situ interviews are often costly, intrusive, and lack comparability across cities, which leads to significant data gaps [3,4]. Recent advances in computer vision and street-level imagery (SLI) offer a promising alternative, as object detection models can be used to identify and count vendors directly from images in a scalable and reproducible manner. However, developing such models requires access to annotated images in which vendors are clearly labelled — a resource that is currently unavailable for the public domain [[5], [6], [7], [8]].

The dataset introduced in this study addresses this gap. Using affordable action cameras mounted on motorcycles, we captured high-resolution SLI frames across diverse commercial corridors in a Latin American city. Each vendor instance was manually annotated with an axis-aligned bounding box and classified according to mobility-based categories: fixed-stall, semi-fixed, or itinerant. Furthermore, privacy-sensitive information such as faces, and license plates were removed.

**Fig. 1:**
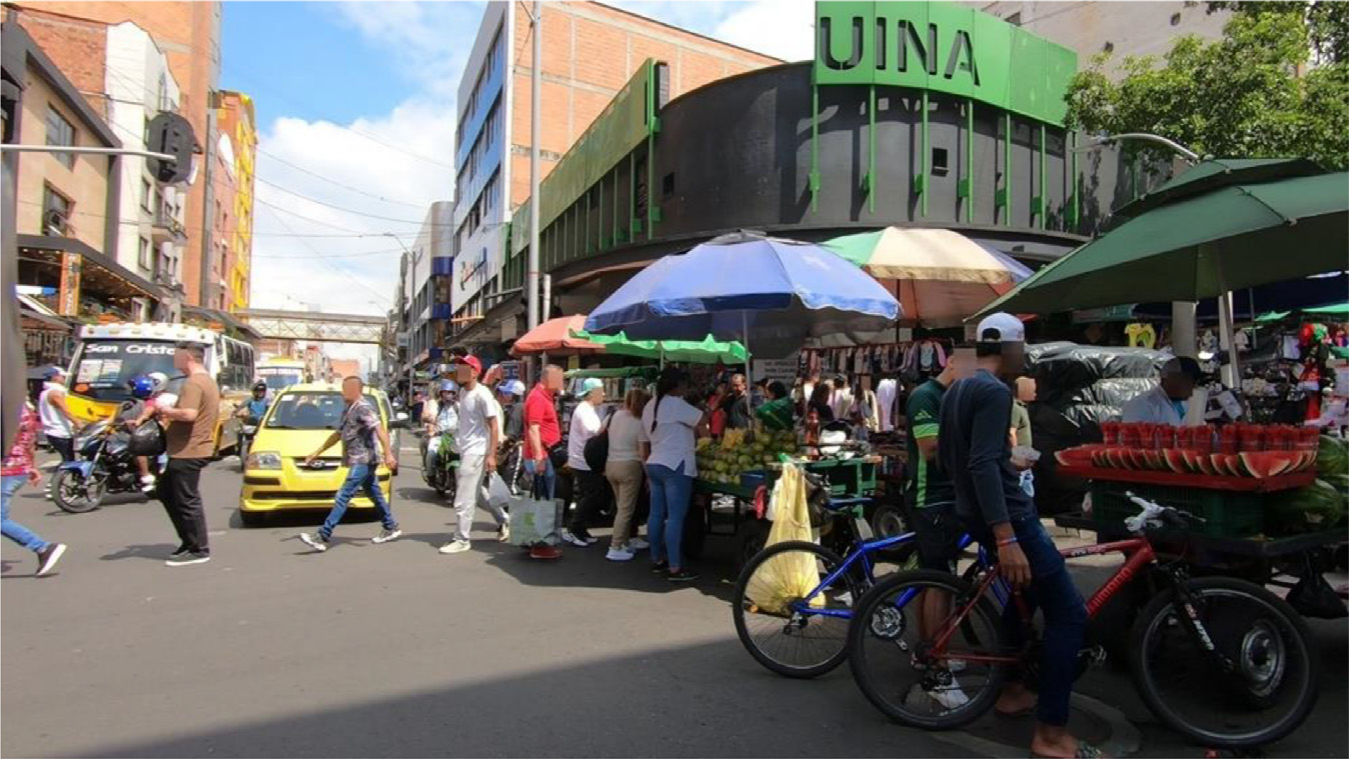
Image format in Raw_Imagery folder.

**Fig. 2:**
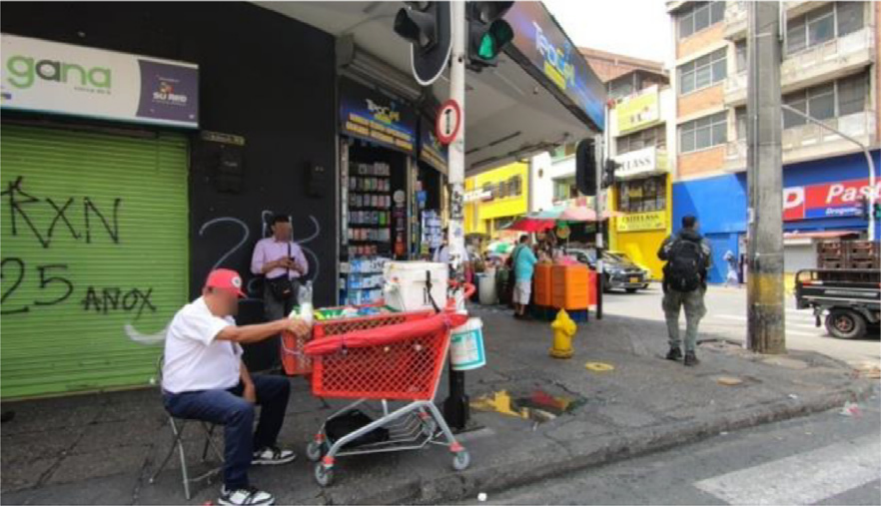
Image and corresponding label format in Dataset folder.

**Fig. 3:**
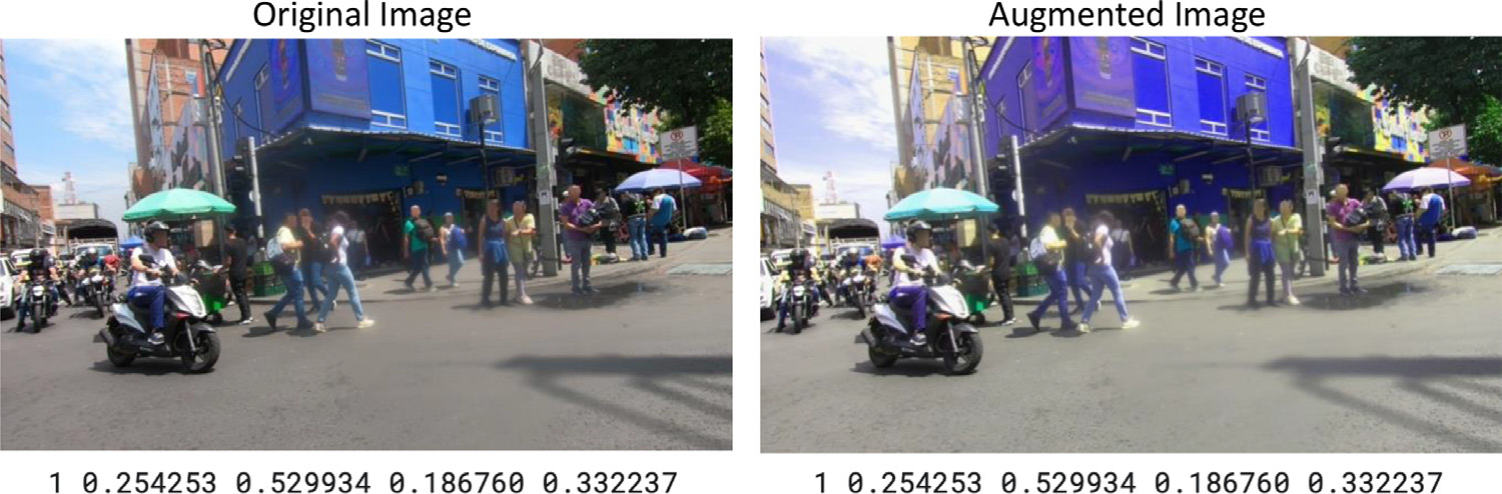
Example of Original and Augmented Image their corresponding Label.

**Fig. 4:**
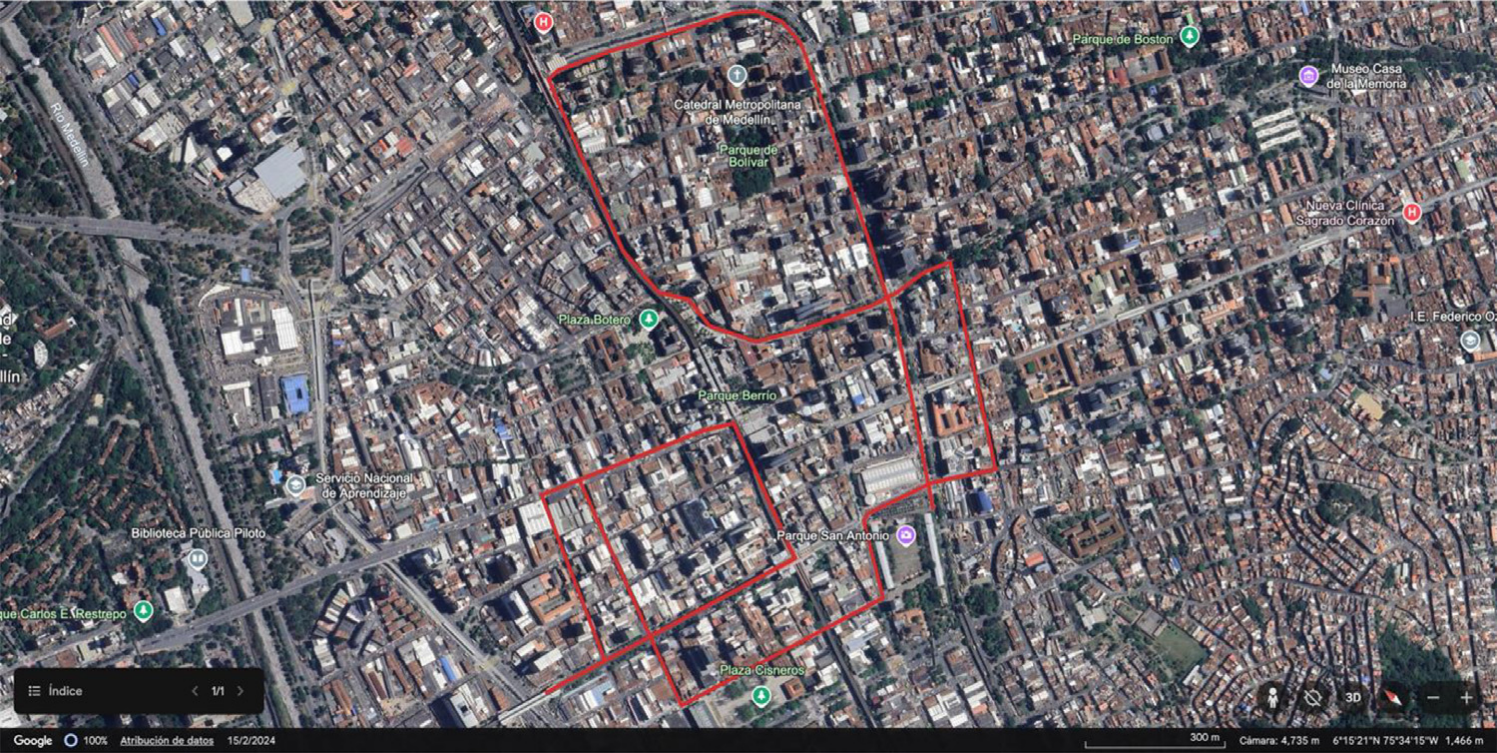
An example of a transect to collect raw videos [9].

By openly releasing this dataset, we aim to provide a resource to enable reproducible, image-based analyses of street-vendor presence (Fig. 1, Fig. 2, Fig. 3, Fig. 4).

## 3 Data Description

The dataset is structured to allow flexibility for modifications, including the application of additional data augmentation techniques [1]. This design facilitates adaptation, expansion, and comparison as required by researchers. The dataset consists of four directories, each of which is described below:

• Raw_Imagery: Comprises the unlabeled and anonymized images extracted from the video. This information enables each researcher to perform their own image labelling process.
• Dataset: Contains image-label pairs. The labels are stored as .txt files In YOLO format (class id x_center y_center width height), each specifying image coordinates of the labeled class within the images. The process of image extraction and labelling is described in the following section.
• Data_augmentation: Contains the augmented images along with their corresponding labels. The applied augmentation methods are described in the next section.
• Low_Resolution_Dataset: It includes all the image-label pairs, but the images are downgraded to a lower resolution in percentage with the aim to speed up a network training process.

The dataset information is summarized in the Table 1 as follows:

**Table 1:** Dataset specifications.

Item	Description
Dataset size	4.63 GB
Video format	.mp4
Image format	.jpg
Image size	2416×1359 px
Image quantity	1397 (original), 2794 (after augmentation)
Labels format	YOLO
Channels	3 bands, RGB
Data Augmentation	Geometric and Spectral
Average memory per image	5Mb

## 4 Experimental Design, Materials and Methods

To detect street vendors, we propose to use the next steps to create a customized, flexible and anonymized dataset, specifically adjusted to meet unique research needs and local environments, thereby improving the applicability of the analysis.

### 4.1 Street-level imagery acquisition

In this paper, side-looking videos were collected using a GoPro action camera mounted on a motorcycle's handlebar. These videos were subsequently processed into street-view images using a Python script, producing one SLI every two seconds of video. The resulting dataset contains 2794 street-level images after imagery augmentation. The data collection process entailed covered 37 km along 10 transects within La Candelaria sector in Medellín, Colombia. Each transect took approximately 30–35 min time, requiring battery swap when depleted. Recordings were carried out during daytime, specifically from 9:00 AM to 12:00 PM. The data acquisition process imposed several challenges inherent to field-based imagery collection. Unfavorable weather conditions, such as rain, occasionally resulted in dirty camera lens, introducing blurriness and visual artifacts in some images. Furthermore, variations in camera settings and lighting across different collection sessions led to inconsistencies in image dimensions and color balance, which complicate subsequent standardization and processing. Additionally, the use of high-resolution imagery represents practical difficulties for storage, computational resources, and analysis due to their large file sizes. Fig. 4 illustrates one of the transects that was taken.

### 4.2 Data processing and privacy preservation

#### 4.2.1 Anonymization of pedestrian and vehicle license plates

To guarantee that every image in the dataset satisfies the requirements of the European Union’s General Data Protection Regulation (GDPR)[10]. we adopted a two-stage anonymization protocol that combines automatic detection with a subsequent manual verification step.

Stage 1 – Automatic anonymization

All street-level images (SLIs) are first processed by an open-source Python pipeline that leverages the YOLOv7-tiny object-detection network. The model is configured to locate (i) pedestrian faces and (ii) vehicle license plates; the corresponding bounding boxes are then automatically blurred with a Gaussian filter. YOLOv7-tiny was selected because of its high inference speed and competitive accuracy, which enable frame-rate processing without specialized hardware [[11], [12]].

Stage 2 – Manual quality control

Automated methods, while efficient, are not infallible. Consequently, every SLI that had passed Stage 1 was visually inspected by at least two members of the research team. During this review we verified that no face, license plate, or other element that could directly or indirectly identify an individual remained visible, in accordance with GDPR [10]. Images that were found to be insufficiently anonymized were (i) re-processed with stricter parameters or (ii) permanently removed from the dataset. This exhaustive inspection ensures that the final corpus contains only images that comply with GDPR provisions on personal data protection.

**Fig. 5:**
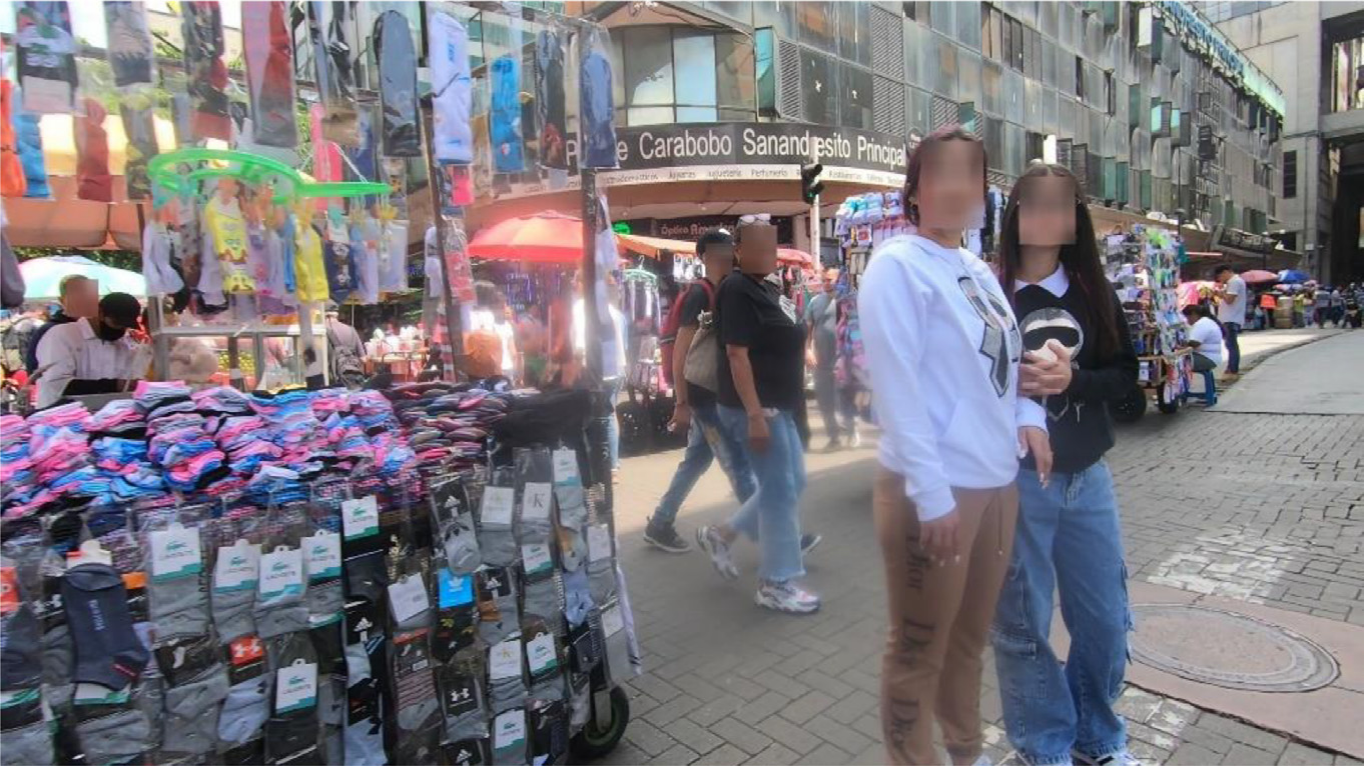
Face detection and blurring.

Fig. 5 shows an example of an SLI before and after the two-stage anonymisation procedure [13].

By combining a state-of-the-art object-detection model with systematic human oversight, we minimize the risk of disclosing personally identifiable information and demonstrate a robust commitment to privacy preservation throughout the data-preparation workflow.

#### 4.2.2 Data annotation

For data annotation, we utilized the open-source tool LabelImg, developed by Tzutalin [14]. This software generates an XML file for each image, which stores the bounding boxes coordinates along with the corresponding class label. Each image was carefully reviewed and manually annotated by drawing axis-aligned rectangles around every street vendor to accurately capture their X–Y location.

Vendors were classified into three mutually exclusive categories based on their spatio-temporal behavior and the stand characteristics:

I. Fixed-stall-vendor – Operates permanently at a single location, with no means of mobility during a day (e.g., kiosks or tables with umbrellas).
II. Semi-fixed vendor – can relocate between trading sessions and temporarily park in different spaces; the infrastructure (e.g., tricycle or hand-pushcart) is purposely built for movement, but remains stationary while sales occur.
III. Itinerant vendor – Continuously moves through streets or public spaces while selling goods carried on small, highly mobile supports such as baskets, backpacks, or supermarket trolleys. Due to their limited infrastructure used, the quantity and dimensions of the products are also restricted. The primary distinction from semi-fixed vendors lies in this reduced storage capacity and light weight load.

To ensure labeling accuracy and consistency across the dataset, all annotated images were subjected to a post-annotation manual review. During this quality control step, the research team re-examined each image to verify that bounding boxes and class labels were correctly assigned according to the defined annotation criteria.

**Fig. 6:**
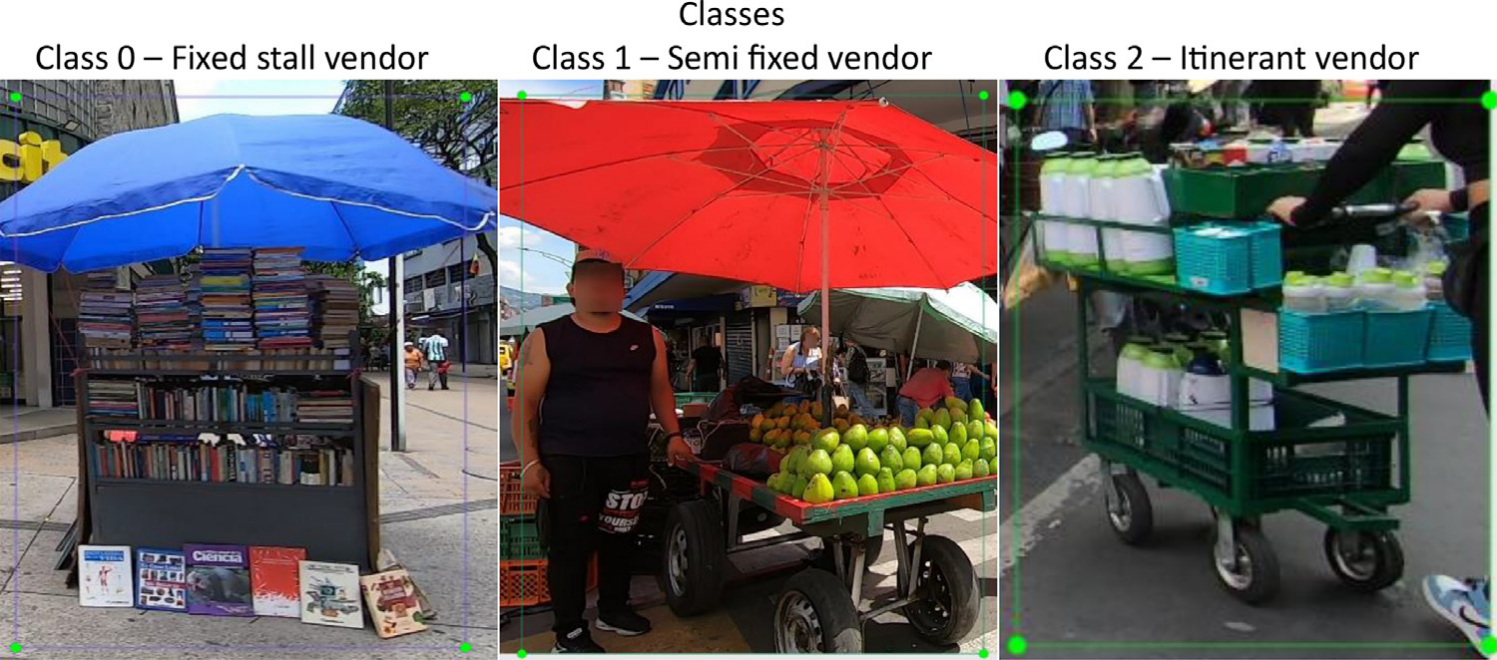
Street vendors for each class.

**Fig. 7:**
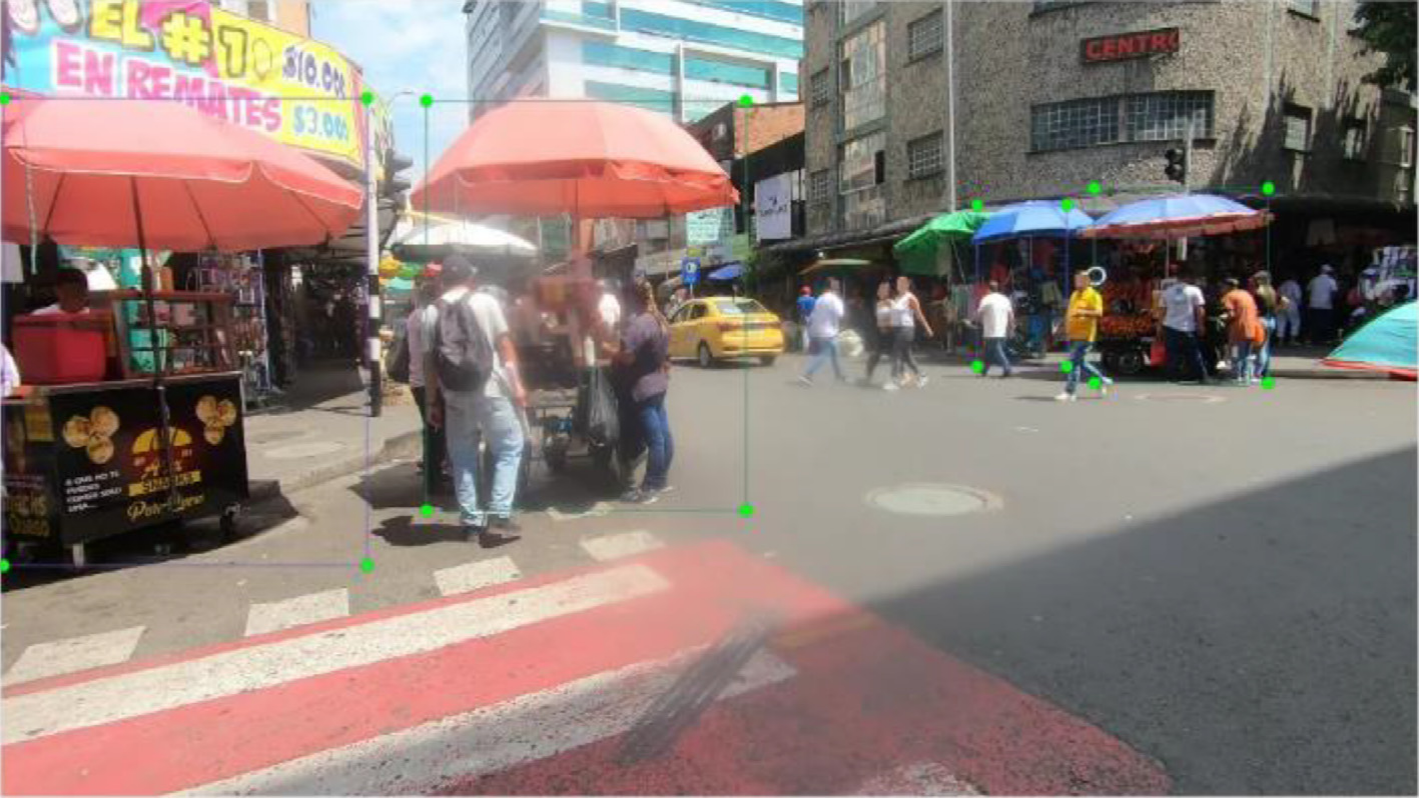
Data Annotation Process.

During pre-processing, images without street vendors were excluded, resulting in a total of 1397 annotated image–label pairs. To augment the dataset, standard geometric and spectral transformations were applied, expanding the collection to 2794 pairs. Representative examples of the three vendor classes are shown in Fig. 6, while Fig. 7 illustrates the annotated bounding boxes generated using LabelImg.

#### 4.2.3 Data augmentation

Data augmentation encompasses a set of techniques to create new samples from existing data. This approach helps to reduce overfitting by increasing the variability of the original dataset. This enhances both the size and quality of the dataset. Other strategies for improving generalization focus on the model architecture, including methods such as dropout, batch normalization, transfer learning, one-shot and zero-shot learning [15]. A particular challenge addressed through this process was the class imbalance, as certain vendor categories had significantly fewer samples than others. To mitigate this issue and ensure a more balanced representation, geometric and spectral transformations were applied to the original dataset [15,16]. However, it is important to note that the original class proportions were intentionally preserved to maintain the natural representativeness of the phenomenon being studied. Specifically, street and semi-fixed vendors are inherently less frequent in real-world scenarios. Artificially balancing the dataset could introduce bias by assigning disproportionate weight to these less common classes, potentially distorting results and compromising the validity of subsequent analyses.

### 4.3 Geometric transformation

Geometric transformations modify images by changing their scale and orientation without altering their fundamental content [15,16]. In this study, we apply rotation, flipping, scaling, and shearing transformations using the Albumentations Library [17].

### 4.4 Spectral transformation

**Fig. 8:**
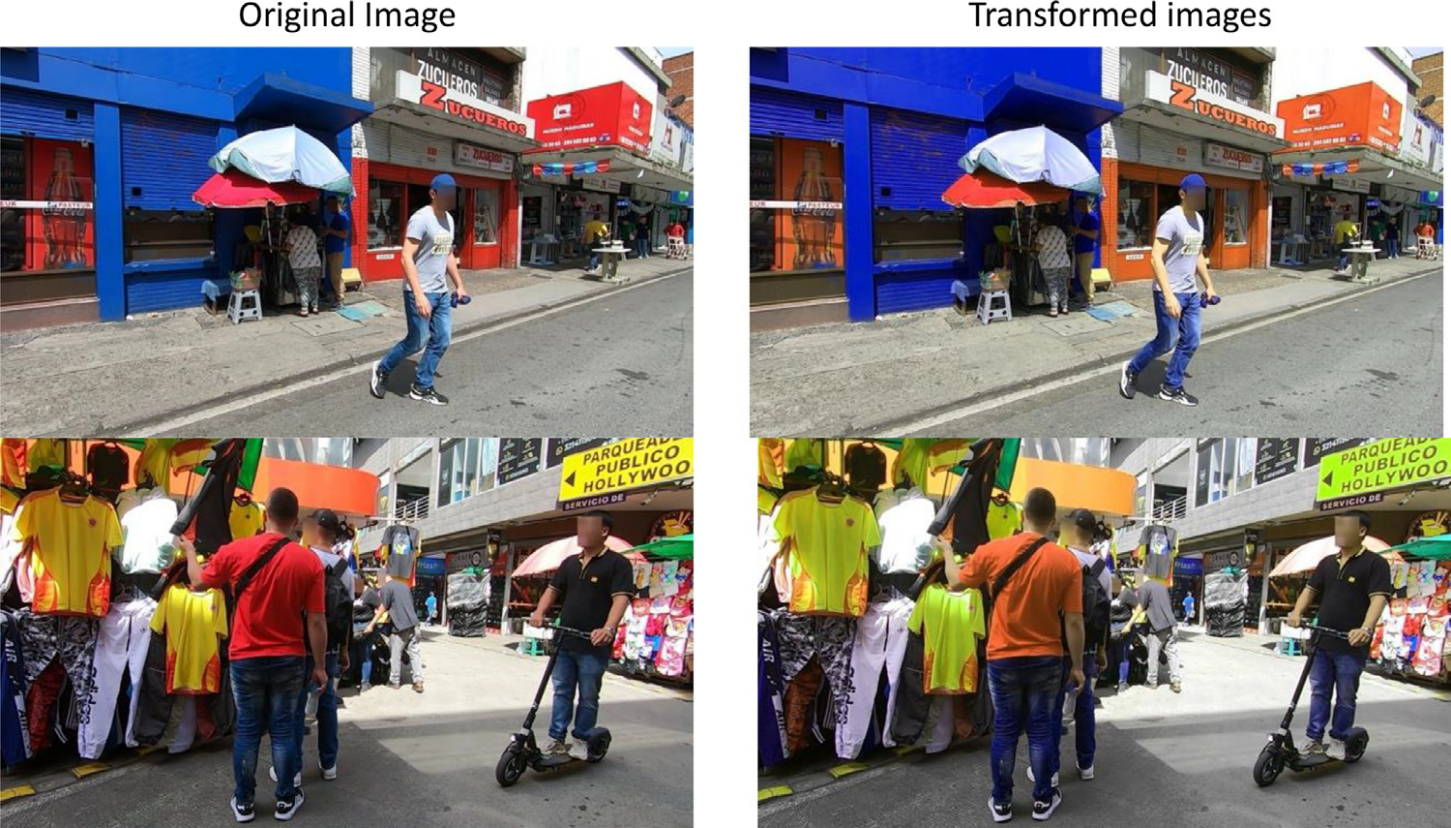
Data Augmentation transformations.

It modifies colour properties within an image like brightness, contrast, saturation, and hue, as well as blur and CLAHE (Contrast Limited Adaptive Histogram Equalization). Fig. 8 shows some samples generated with the data augmentation transformation.

### 4.5 Resolution downgrade

To speed the training process up, we produced a separate dataset by reducing the resolution of the images by 25 %. Lowering the resolution decreases the size of each image, which directly reduces the training time of the model by requiring less computational power and memory, this approach may also limit the model's ability to learn fine-grained features, potentially affecting its overall accuracy and generalization capability. Therefore, it is crucial to carefully evaluate a trade-off and select the dataset that best suits the specific needs of the task. A very simple Python script is provided to downgrade images resolution to a custom percentage.

### 4.6 Comparative context with related studies

To situate the methodological contribution of the Street Vendor-SLI dataset, we reviewed recent studies that developed their own datasets for the detection or analysis of street vendors using street-level imagery and machine learning. While these datasets are not publicly available, their approaches provide relevant context regarding common design choices and detection strategies.

Table 2 summarizes the main characteristics of these works and allows for a comparison with the Street-level Imagery dataset. Unlike the studies listed, which primarily rely on images extracted from third-party APIs (such as Google Street View), our dataset was built entirely from original imagery collected in the field. This distinction not only enables open-access distribution, but also ensures higher control over image quality, vendor visibility, and privacy compliance. In addition, Street-level Imagery dataset introduces a three-class annotation scheme based on vendor mobility — a novel contribution not found in previous work.

**Table 2:** Comparative with related studies.

Article	Description
**Steet-level Imagery Dataset for the Detection of Informal Vendors in Urban Environment.** (K. Garcia Jaimes, J. Ballesteros, and J. W. Branch Bedoya – 2025) [1]	Introduces a novel, fully open-access dataset of 2794 high-resolution images, captured with field-deployed action cameras in a real urban environment. Includes manual annotations with bounding boxes and a three-class vendor typology based on mobility (fixed-stall, semi-fixed, itinerant). Ensures full GDPR compliance through automated and manual anonymization.
**Detecting the city-scale spatial pattern of the urban informal sector by using street view images: A street vendor massive investigation case** *(Yilun Liu, Yuchen Liu – 2022) [**3**]*	Introduces the SIPSI methodology, applying deep learning to Google Street View images to analyze vendor clustering across socioeconomic zones (non-public dataset).
**Predicting the heat map of street vendors from pedestrian flow through machine learning** *(Shou, X., et al. – 2021)* [18]	Uses pedestrian flow and semantic segmentation with GANs to predict vendor locations, producing heatmaps based on image-derived traffic patterns.
**Uncovering commercial activity in informal cities** *(Straulino, D., et al. – 2022)* [19]	Evaluates object detection models (YOLO, SSD) for detecting informal commercial activity using street-level imagery from external sources.
**Street Vendor Detection: Helping municipalities make decisions with actionable insight** *(Ọgba, H. N., & Tahir, A. – 2021)* [20]	Trains a MobileNet SSD-based object detector on vendor imagery to support municipal policy, using non-public and unspecified image sources.

## Limitations

This dataset has some limitations. First, its size is relatively small, comprising <3000 images, which might limit the generalization of models. Additionally, the dataset is subject to data biases, as all images were collected from a single study area, potentially restricting its applicability to other regions. Quality issues are also present in some images. For example, blurriness may result from a dirty camera lens caused by bad weather conditions, such as rain, which can introduce visual artifacts. Variations in camera configurations during data collection further contribute to inconsistencies in image dimensions and color differences, complicating standardized processing. Finally, the high resolution of the images results in large file sizes, which can pose challenges for storage, computational processing, and analysis. These aspects should be carefully considered when utilizing this dataset in research or practical applications.

## Ethics Statement

All the images in the dataset have been fully anonymized, with all personal information removed to comply with GDPR guidelines for personal data protection. These guidelines require safeguarding any information that can directly or indirectly identify an individual, including names, email addresses, location data, biometric details, and more. To ensure privacy, we blur identifiable features such as pedestrian faces and vehicle license plates in every image. The dataset permits use, sharing, adaptation, distribution, and reproduction in any medium or format if appropriate credit is given to the author(s)

## Credit Author Statement

**Keyla Garcia Jaimes:** Conceptualization, Data curation, Software, Writing- Original draft preparation; **John R. Ballesteros:** Writing- Reviewing and Editing, Project administration, Supervision. **John W. Branch:** Supervision.

## Data Availability

ZenodoSteet-level Imagery Dataset for the Detection of Informal Vendors in Urban Environment (Original data).
